# A Combination of *Curcuma longa* and Diazepam Attenuates Seizures and Subsequent Hippocampal Neurodegeneration

**DOI:** 10.3389/fncel.2022.884813

**Published:** 2022-06-14

**Authors:** Chirlene Pinheiro Nascimento, Luan Oliveira Ferreira, Alex Luiz Menezes da Silva, Ana Beatriz Nardelli da Silva, Joao Cleiton Martins Rodrigues, Leonan Lima Teixeira, Julianne Elba Cunha Azevedo, Daniella Bastos de Araujo, Akira Otake Hamoy, Beatriz Holanda Gonçalves, Brenda Hosana De Oliveira Coelho, Dielly Catrina Favacho Lopes, Moisés Hamoy

**Affiliations:** ^1^Laboratory of Pharmacology and Toxicology of Natural Products, Institute Biological Science, Federal University of Pará, Belém, Brazil; ^2^Laboratory of Experimental Neuropathology, João de Barros Barreto University Hospital, Federal University of Pará, Belém, Brazil

**Keywords:** *Curcuma longa*, seizure, diazepam, neurodegenaration, hippocampus

## Abstract

Epilepsy is one of the most common neurological disorders, which occurs due to the instability in the inhibitory and excitatory synaptic transmissions in the brain. However, many patients develop resistance to the available drugs, which results in cell degeneration caused due to inadequate control of the seizures. Curcumin, *Curcuma longa*, is known to be effective for the treatment of organic disorders and may prevent seizures, reduce oxidative stress, and decrease brain damage. Given this, the present study evaluated the antiepileptic effects of *C. longa* in comparison with both the diazepam and the combined application of these two substances, in terms of their effects on the brain activity and the potential histopathological changes in the hippocampus. This study used male Wistar rats (age: 10–12 weeks; weight: 260 ± 20 g), which were pretreated for 4 days with either saline, *C. longa*, diazepam, or *C. longa* + diazepam; and on the fifth day, pentylenetetrazol (PTZ) was administered to induce the seizure. In the *C. longa* group, a significant increase was observed in the latency of the onset of seizure-related behavior. Surprisingly, however, the combined treatment resulted in the best control of the seizure-related behavior, with the greatest latency of the onset of spasms and isolated clonic seizures. This group also obtained the best results in the electroencephalographic trace and seizure control, with a reduction in the frequency and amplitude of the spike-waves. In the saline group, PTZ significantly reduced the number of cells present in the CA1 and CA3 regions of the hippocampus, while the combined treatment obtained the best results in terms of the preservation of the neuron-like cells. These findings indicate that *C. longa* may contribute to the control of both seizures and the cell damage induced by PTZ, and that its association with diazepam may be a potentially effective option for the treatment of epilepsy in the future.

## Introduction

Epilepsy is one of the most common neurological disorders, affecting approximately 0.7% of the human population worldwide (Fiest et al., 2017). This condition occurs as a result of instability in both the inhibitory synaptic transmission in the brain, which reduces the transmission mediated by the GABA receptors, and the excitatory transmission, which increases the glutamatergic signaling. The available antiepileptic drugs (AEDs) thus act through two pathways, i.e., by either (1) potentiating the inhibitory mechanisms or (2) reducing the excitatory signaling (Sultana et al., 2021).

Although effective medication is available for the control of seizures, approximately one-third of the patients do not respond satisfactorily to the treatment, based on trials involving at least two different AEDs (either individually or in combination) that fail to impede seizures (of all types) in the patients (Sierra et al., 2015; Kalilani et al., 2018). Thus, it is essential to identify additional potential treatments that act on the underlying mechanisms that determine the seizures and have minimal side effects (Sultana et al., 2021).

Epilepsy can also cause neuronal damage in electrically sensitive regions, such as the hippocampus, which means that prolonged seizure activity may lead to increased production of reactive oxygen species, oxidative stress, and mitochondrial dysfunction, eventually leading to severe cerebral damage (Dillioglugil et al., 2010). The oxidative stress and mitochondrial dysfunction provoked by epilepsy disrupt the homeostasis of the intracellular environment, resulting in neuroexcitability and cell death. Oxidative stress damages the mitochondrial respiratory chain and leads to the excessive production of reactive oxygen species, which accumulates to the point of inhibiting the activity of the mitochondrial respiratory chain, eventually resulting in neurodegeneration (Chang and Yu, 2010).

One of the regions most affected by epilepsy is the hippocampus due to its electrical vulnerability. The sensitivity of the hippocampus is due to the presence of a large number of GABAergic neurons in the deeper regions of the dentate gyrus. Glutamate is the principal excitatory neurotransmitter in the hippocampus, and during periods of hyperexcitability, i.e., epilepsy, convulsions occur, which may result in cell death, primarily in the regions rich in glutamatergic receptors, such as the CA1 and CA3 regions (Casillas-Espinosa et al., 2020).

Curcumin is a principal biologically active compound extracted from *Curcuma longa*, which has been shown to be effective in the treatment of a number of organic disorders (Witkin and Li, 2013; Pricci et al., 2020). Previous studies in several countries have demonstrated the therapeutic value of *C. longa* as an antioxidant, anti-inflammatory agent, or gut microbiome modulator in *in vitro*, *in vivo*, and clinical trials in humans (Yu et al., 2013; Thumann et al., 2019; Rodrigues et al., 2021). Mehla et al. (2010) found that curcumin is effective as an anticonvulsant, with the potential to prevent seizures, reduce oxidative stress, and decrease brain damage. These findings indicate that curcumin may have potent antiepileptic effects, in particular by delaying the onset of seizures, although the exact mechanisms through which it achieves these results are still unclear (Mehla et al., 2010).

In this context, the present study evaluated the antiepileptic effects of *C. longa* in relation to brain activity in comparison with diazepam (DZP) and the combined treatment (*C. longa* + DZP), based on the decomposition of brain waves using electroencephalograms (EEGs) in a pentylenetetrazol (PTZ)-induced seizure model. The effects of the different treatments were also evaluated in terms of the results of an electromyogram (EMG) and histopathological changes in the hippocampus.

## Materials and Methods

### Animals

The present study used male Wistar rats (*n* = 72 animals) aged 10–12 weeks and weighing 260 g (± 20 g). These animals were housed in standard cages in a controlled environment (22 ± 2°C; 12/12 h light/dark cycle, 55 ± 10% relative humidity) with *ad libitum* access to food and water. The experimental procedures were approved by the relevant Brazilian federal agencies and were in accordance with the Brazilian National Council for the Control of Animal Experimentation and the Ethics Committee on Use of Animals of the Biological Sciences Institute at the Federal University of Pará (CEUA/UFPA no. 9149220321). The data presented here were also collected in compliance with the ARRIVE (Animal Research: Reporting *In Vivo* Experiments) guidelines. All necessary precautions were taken to prevent animal suffering and distress.

### Experimental Design

**FIGURE 1:**
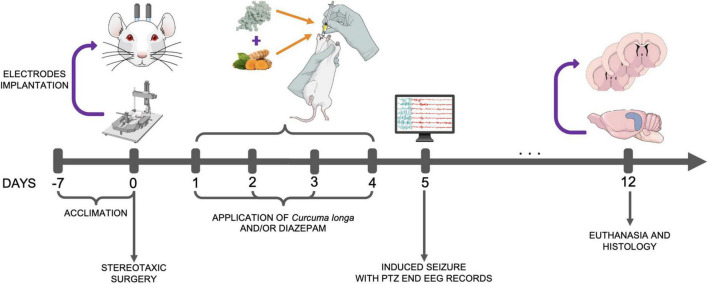
Experimental design. PTZ, pentylenetetrazol; EEG, electroencephalograph.

The animals in this study were maintained in the research facility for at least 7 days prior to the experiment, for adaptation and acclimation (Figure 1), with the electrodes being implanted in the cortex 1 day prior to the application of the treatments. During the experiment, the rats were pretreated for 4 days with either saline, *C. longa*, DZP, or *C. longa* + DZP *via* the orogastric route (gavage) at 24-h intervals. On the fifth day (24 h after the last application), seizures were induced by a single dose of PTZ, intraperitoneally (Agarwal et al., 2013), with electroencephalographic and EMG records being collected over the subsequent 15 min. The rats were monitored for the subsequent 7 days (follow-up) prior to being euthanized. The brain was then extracted, sectioned, and stained with cresyl violet for cell counting. All these procedures were conducted strictly between 08:00 and 11:00 a.m.

The animals were divided into eight groups (each containing nine animals): (i) saline + saline (SAL); (ii) *C. longa* (CL) + saline; (iii) DZP + saline; (iv) CL/DZP + saline; (v) SAL + PTZ; (vi) CL + PTZ; (vii) DZP + PTZ; (viii) CL/DZP + PTZ. The seizure behavior was recorded after the application of PTZ.

### Drugs

In addition to the two drugs evaluated in the present study, three types of anesthetics were also used for handling the rats. *C. longa* was applied in the form of purified pharmaceutical grade curcumin, supplied as 250-mg capsules containing 50 mg of curcumin together with excipients (Aché Laboratórios Farmacêuticos S.A., Brazil). The curcumin was administered *via* oral gavage at a dose of 80 mg/kg (Akula and Kulkarni, 2014); while the DZP, 10 mg/2 ml (União Química, Embu-Guaçu, SP, Brazil), was administered at a dose of 5 mg/kg (V.O.). Three different types of anesthetic were used in the present study. Ketamine hydrochloride was obtained from the Köing Laboratory (Santana de Parnaíba, SP, Brazil) and xylazine hydrochloride was acquired from the Vallée Laboratory (Montes Claros, MG, Brazil), while the local anesthetic lidocaine, which was used to implant the electrodes, was obtained from the Hipolabor Laboratory (Sabará, MG, Brazil). The PTZ was obtained from Sigma Chemical Co. (St. Louis, MO, United States).

### Electroencephalographic Recordings and Data Analyses

The EEGs were recorded as described by Estumano et al. (2019). For this, the animals were anesthetized and placed in a stereotaxic apparatus for the implantation of stainless-steel electrodes (exposed tip 1.0 mm in diameter) on the dura mater above the pre-frontal cortex at the bregma coordinates − 0.96 mm and ± 1.0 mm lateral, and were fixed with dental acrylic cement. The data were registered with the help of the electrodes using a digital data acquisition system composed of a high impedance amplifier (Grass Technologies, P511, United States), an oscilloscope (Protek, 6510, United States), and a data acquisition and digitalization board (National Instruments, Austin, TX, United States). Data were collected continuously at 1 kHz, at a low pass of 3 kHz, and a high pass of 0.3 Hz. During the recording sessions, the animals were confined to acrylic boxes (20 cm × 45 cm × 15 cm), and the EEG activity was recorded for 15 min immediately after the application of the PTZ or saline solution. The records collected using the digital data acquisition system were analyzed offline. The analyses were run at frequencies of up to 40 Hz, and then split into four bands, that is, the delta (1–4 Hz), theta (4–8 Hz), alpha (8–12 Hz), beta (12–28 Hz), and gamma (28–40 Hz) bands (Aminov et al., 2017).

### Description of the Seizure-Related Behavior

The behavior of the animals was monitored during the seizures and compared with the latency patterns of the behaviors observed in the PTZ group. Latency was measured in relation to the onset of the following behaviors: (i) generalized tremor; (ii) spasms of the forelimbs; (iii) isolated clonic seizures with no loss of the posture reflex; (iv) generalized clonic seizures with transient loss of the posture reflex; and (v) tonic-clonic seizures with total loss of the posture reflex.

### Electromyographic Recordings

Electrodes were implanted in parallel to the masseter muscle, 5 mm above their point of insertion in the jaw to record the muscle activity during seizures in the PTZ groups. As for the EEG, the data were recorded for 15 min (Santos et al., 2021).

### Nissl Staining and Cells Count

Once euthanized, the rats were perfused transcardially with phosphate-buffered saline (PBS, pH 7.4) at 4°C, followed by 4% formaldehyde (pH 7.4). The brain was extracted post-reperfusion, fixed in 4% formaldehyde for 72 h, cryoprotected in 30% sucrose for 24 h, and then cut into serial coronal sections (40 μm) and stained with Nissl (0.3% cresyl violet acetate). The number of cells of six coronal sections of the hippocampus (CA1 and CA3) of each rat were counted to provide a mean count for each group (*n* = 9 rats per group). The cell counts were based on the inspection of a field of 50 μm × 50 μm in each region (Wang et al., 2021). The counted (treatment-blind) cells were observed using the ImageJ digital imaging software (NIH, Bethesda, MD, United States).

### Statistical Analyses

The normality of the data variances was verified using the Kolmogorov–Smirnov test. All the data are presented as the mean and standard deviation (SD), and the *F* and *p*-values are included where pertinent. A *p* < 0.05 significance level was considered for all the analyses. The significance of differences between the pairs of groups was verified using Student’s *t-*test, while the variation among three or more groups was evaluated using an Analysis of Variance (ANOVA), either one-way or two-way, followed by Tukey’s test for pairwise multiple comparisons. The analyses were run in GraphPad Prism, version 9 (Graph-Pad Software Inc., San Diego, CA, United States).

## Results

### The Combination of *Curcuma longa* and Diazepam Prevents the Progression of Seizure Behavior

The behavior of the rats was assessed to determine the evolution of the seizures (Table 1). The animals pretreated with saline that received PTZ progressed quickly to tonic-clonic seizures with the loss of the postural reflex after a mean interval of less than 5 min. Latency prior to the onset of seizure increased significantly in the group pretreated with *C. longa*, although the evolution to tonic-clonic seizure with loss of postural reflex was not interrupted.

**TABLE 1:** Description of the seizure-related behavior of animals treated with *Curcuma longa* and/or diazepam.

	Generalized tremor	Spasms of the forelimbs	Isolated clonic seizures without loss of posture reflex	Generalized clonic seizures with transient loss of posture reflex	Tonic-clonic seizures with loss of posture reflex
SAL + PTZ	47.44 ± 4.851	61.11 ± 8.007	72.11 ± 10.65	146.1 ± 34.58	248.9 ± 130.2
CL + PTZ	72.56 ± 14.30	117.8 ± 38.27	180 ± 50.58*	437.7 ± 45.06*	708.9 ± 113.7*
DZP + PTZ	323.4 ± 41.39*^#^	447.9 ± 84.84*^#^	589.3 ± 55.07*^#^	–	–
CL/DZP + PTZ	322.3 ± 88.43*^#^	622.3 ± 96.36*^#@^	838.7 ± 83.14*^#@^	–	–
*F*-value and *p*-value	*F*_(3, 32)_ = 85.35 *p* < 0.0001	*F*_(3, 32)_ = 153.0 *p* < 0.0001	*F*_(3, 32)_ = 363.9 *p* < 0.0001	*F*_(3, 32)_ = 474.9 *p* < 0.0001	*F*_(3, 32)_ = 134.7 *p* < 0.0001

The data are expressed as the mean ± SD (n = 9 animals per group: *p < 0.05 vs. PTZ, ^#^p < 0.05 vs. CL + PTZ, and ^@^p < 0.05 vs. DZP + PTZ). PTZ, pentylenetetrazol; CL, Curcuma longa; DZP, diazepam.

The group pretreated with DZP that received PTZ presented a greater latency to the onset of seizures in comparison with the *C. longa* group, in addition to the stabilization of the symptoms, given that the rats presented only isolated clonic seizures with no loss of the postural reflex. Surprisingly, the combined pretreatment (*C. longa* + DZP) resulted in even better control of the seizure-related behavior, in comparison with the DZP + PTZ group, with the greatest latency to the onset of the spasms and only isolated clonic seizures. These results indicate that the association of *C. longa* and DZP may provide effective control and prevent the evolution of the seizure.

### The Pentylenetetrazol-Induced Seizure Is Attenuated in the Electroencephalogram by the Combined Use of *Curcuma longa* and Diazepam

**FIGURE 2:**
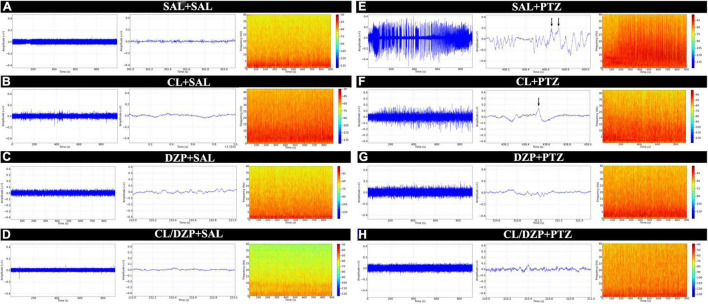
Electroencephalographic recordings of animals submitted to the PTZ-induced seizure model and treated with *Curcuma longa* and/or diazepam. The linear trace was obtained by electroencephalography (left). Representative 1 s sequence of the EEG trace (center). Spectrogram frequency (right). **(A)** Animals that received no treatment (SAL-SAL). **(B)** Animals pretreated with *Curcuma longa* but not injected with PTZ (CL-SAL). **(C)** Animals pretreated with diazepam but not injected with PTZ (DZP-SAL). **(D)** Animals pretreated with *Curcuma longa* + diazepam not injected with PTZ (CL/DZP-SAL). **(E)** Animals pretreated with saline and injected with PTZ (SAL-PTZ). **(F)** Animals pretreated with *Curcuma longa* and injected with PTZ (CL-SAL). **(G)** Animals pretreated with diazepam and injected with PTZ (DZP-PTZ). **(H)** Animals pretreated with *Curcuma longa* + diazepam and injected with PTZ (CL/DPZ-PTZ). SAL, saline; CL, *Curcuma longa*; DZP, diazepam; PTZ, pentylenetetrazol.

The EEGs were first obtained from the four saline groups (i–iv), that is, the animals that were pretreated with saline, *C. longa*, DZP, and *C. longa* + DZP, respectively, and then received saline on the fifth day (+ SAL). This provided a baseline for the verification of the possible effects of the pretreatment on brain activity. The animals pretreated with saline (group i) had amplitudes below 0.02 mV (Figure 2A), and the spectrogram reveals energy concentrations of below 10 Hz. None of the animals of the other groups (ii–iv) presented any significant difference in the brain activity (Figures 2B–D) in comparison with the control (i), which indicates that none of the pretreatments alter this activity.

In contrast, group v (SAL + PTZ) presented significant changes in the EEG trace, with peaks of amplitude of over 0.3 mV, and activity characterized by constant levels of spike-waves with a high frequency and amplitude (black arrow, Figure 2E). In group vi (CL + PTZ), amplitude varied up to 0.2 mV, while the frequency and amplitude of the spike-waves decreased (black arrow, Figure 2F). In group vii (DZP + PTZ), the changes in the EEG trace were less intense than in groups v and vi, that is, close to 0.1 mV (Figure 2G), which indicates control of the seizure. Finally, the combined pretreatment (CL/DZP + PTZ) obtained the best results in terms of seizure control, with an amplitude of 0.08 mV, and a reduction in the frequency and amplitude of the spike-waves (Figure 2H).

**FIGURE 3:**
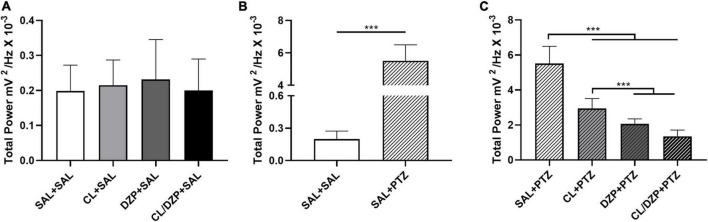
Total linear brainwave power recorded by electroencephalography. **(A)** Quantitative distribution of the total linear power of the brainwaves of the animals that received saline on the fifth day. **(B)** Quantitative distribution of the animals that were pretreated with saline and received saline or pentylenetetrazol on the fifth day. **(C)** Quantitative distribution of the total linear power of the brainwaves of the animals that received pentylenetetrazol on the fifth day. The data are expressed as the mean ± SD (*n* = 9 per group); ***p < 0.001. SAL, saline; CL, *Curcuma longa*; DZP, diazepam; PTZ, pentylenetetrazol.

In addition, the total power did not vary significantly among the saline groups, i.e., groups i–iv [*F*_(3, 32)_ = 0.2671; *p* = 0.8486; Figure 3A]. The administration of PTZ to the saline group (v) resulted in a significant increase in the total power in comparison with group i (SAL + SAL: 0.1985 ± 0.0740 mV^2^/Hz × 10^–3^ vs. SAL + PTZ: 5.509 ± 0.9856 mV^2^/Hz × 10^–3^; *p* < 0.0001; Figure 3B). Significant variation [*F*_(3, 32)_ = 78.75; *p* < 0.0001; Figure 3C] was also found among the other PTZ groups (vi–viii), with all the different pretreatments reducing the total power of the PTZ-induced seizures. The mean total power of group vi (CL + PTZ) was 2.942 ± 0.5694 mV^2^/Hz × 10^–3^, which was significantly lower (*p* < 0.0001) than the PTZ group (v). The mean total power of group vii (DZP + PTZ) was 2.066 ± 0.2846 mV^2^/Hz × 10^–3^, significantly lower than that recorded for either group v (*p* < 0.0001: DZP + PTZ vs. SAL + PTZ) or vi (*p* = 0.0239: DZP + PTZ vs. CL + PTZ). However, the combined treatment (CL/DZP) resulted in the lowest total power of all (1.348 ± 0.3624 mV^2^/Hz × 10^–3^), which was significantly lower than that recorded for the groups v–vii (*p* < 0.0001 in both cases), indicating that this treatment is the most effective one for the control of PTZ-triggered seizures.

### The Association of *Curcuma longa* and Diazepam Reduced Bandpower in the Low-Frequency Brainwaves

**FIGURE 4:**
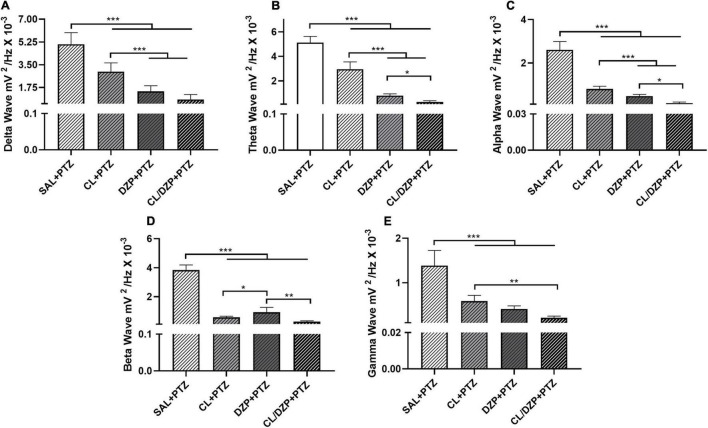
Relative bandpower of the brainwaves (1–40 Hz) of the animals that received pentylenetetrazol on the fifth day (PTZ groups). Quantitative electroencephalographic data on the relative bandpower of the **(A)** delta waves; **(B)** theta waves; **(C)** alpha waves; **(D)** beta waves; **(E)** gamma waves. The data are expressed as the mean ± SD (*n* = 9 animals per group); *p < 0.05, **p < 0.01, ***p < 0.001. SAL, saline; CL, *Curcuma longa*; DZP, diazepam; PTZ, pentylenetetrazol.

The decomposition of the brainwaves was analyzed only for the PTZ groups (v–viii). In the case of the low-frequency waves, a significant increase (group v) was recorded in the bandpower of the delta waves [*F*_(3, 32)_ = 80.49; *p* < 0.0001; Figure 4A]. The animals that received PTZ presented brainwave patterns consistent with disorganized brain activity. However, pretreatment with *C. longa* (group vi) attenuated the effects of PTZ significantly (*p* < 0.0001 vs. SAL + PTZ), which indicates beneficial properties for the reduction of seizures. The attenuation of the delta waves in the two DZP groups (vii and viii) was also significantly greater in comparison with the animals pretreated only with *C. longa* (*p* < 0.0001 for DZP + PTZ and CL/DZP + PTZ vs. CL + PTZ).

A similar pattern was observed in the case of the theta waves (Figure 4B), with significant attenuation in the pretreatment groups (vi–viii) in comparison with the saline + PTZ group [*F*_(3, 32)_ = 275.0; *p* < 0.0001]. While *C. longa* alone significantly mitigated the effects of PTZ on the brain (*p* < 0.0001), the pretreatment with DZP (groups vii and viii) was significantly more effective (*p* < 0.0001).

The administration of PTZ also altered the alpha bandpower (Figure 4C) significantly [*F*(3, 32) = 244.8; *p* < 0.0001]. While all the different pretreatments had significant beneficial effects (*p* < 0.0001, for all comparisons with SAL + PTZ), CL/DZP was the most effective (CL + PTZ vs. CL/DZP + PTZ: *p* < 0.0001; DZP + PTZ vs. CL/DZP + PTZ: *p* = 0.0134), which indicates, once again, that the combination of *C. longa* and DZP provides better control than each compound on its own.

Similar results were also obtained for the higher-frequency waves, that is, the beta and gamma waves. As in the case of the alpha wave, all the different pretreatments had a positive effect on the beta wave (Figure 4D), thus reducing the oscillations significantly [*F*_(3, 32)_ = 388.0; *p* < 0.0001]. In this case, however, the *C. longa* treatments (CL or CL/DZP) reduced bandpower significantly more than the group that was pretreated only with DZP (CL + PTZ vs. DZP + PTZ: *p* = 0.0264; CL/DZP + PTZ vs. DZP + PTZ: *p* < 0.01). This indicates that *C. longa* may be especially beneficial for seizure control, in terms of the mechanisms that trigger the beta waves.

A similar pattern was recorded in the case of the gamma wave (Figure 4E), where all the pretreatments reduced the gamma wave bandpower significantly [*F*_(3, 32)_ = 69.62; *p* < 0.0001]. While the combined application of *C. longa* and DZP provided a better control for seizures than *C. longa* alone (*p* = 0.0008), it was no different from pure DZP (*p* = 0.1202).

### *Curcuma longa* Relieves Muscle Contraction in Pentylenetetrazol-Induced Seizure

**FIGURE 5:**
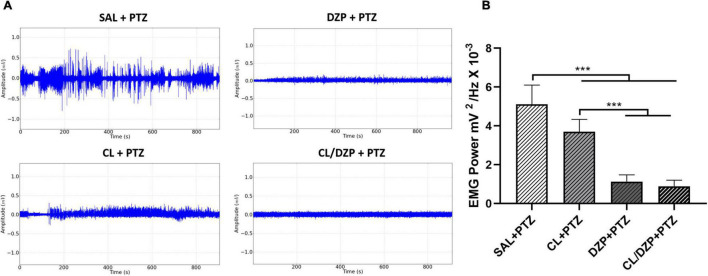
Surface electromyographic recordings of PTZ-induced seizures pretreated with *Curcuma longa* and/or diazepam. **(A)** Electromyographic linear trace. **(B)** Quantitative linear distribution of the power of the muscle contraction. The data are expressed as the mean ± SD (*n* = 9 per group); ***p < 0.001. SAL, saline; CL, *Curcuma longa*; DZP, diazepam; PTZ, pentylenetetrazol.

As orofacial movement (chewing) is a diagnostic trait of PTZ-induced seizures, conjugated electrodes were implanted in the masseter muscle to evaluate its activity during the seizures. Following the application of PTZ, the seizures caused intense muscle contractions, with oscillations in amplitude of up to 0.5 mV in the electromyographic trace (Figure 5A). However, pretreatment with *C. longa* relieved the muscle contractions during the seizure, as revealed by a reduction and stabilization of this amplitude, and a significant reduction in the total power [*F*_(3, 32)_ = 93.35; *p* < 0.0001; SAL + PTZ vs. CL + PTZ: *p* = 0.0003; Figure 5B]. The use of DZP also resulted in intense myorelaxation, with a significant reduction in the total power in the EMG (Figure 5B; *p* < 0.0001). In the combined pretreatment (CL/DZP), the trace was less altered (Figure 5A), with significant improvement in the EMG in comparison with the SAL + PTZ group (Figure 5B, *p* < 0.0001), but not in comparison with the group treated with DZP alone (*p* = 0.8498).

### *Curcuma longa* Decreases the Hippocampal Cell Apoptosis After Pentylenetetrazol-Induced Seizure

**FIGURE 6:**
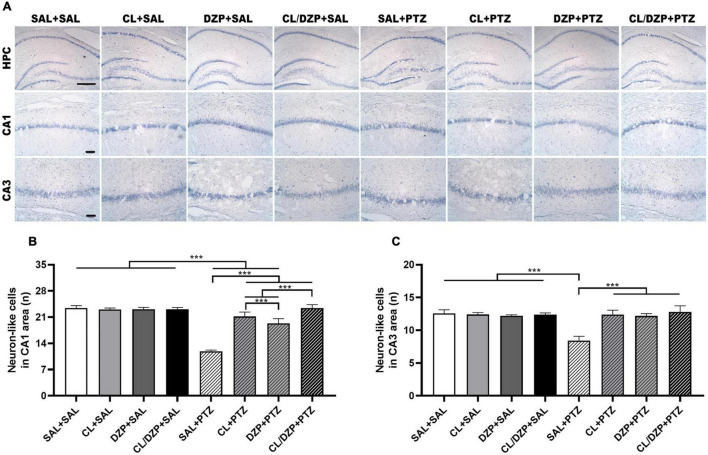
Nissl staining of the rat hippocampus in PTZ-induced seizures pretreated with *Curcuma longa* and/or diazepam. **(A)** Representative images. **(B)** Quantitative data on the number of neuron-like cells in the CA1 region. **(C)** Quantitative data for the number of neuron-like cells in the CA3 region. Data are presented as the mean ± SD (*n* = 9 per group); ***p < 0.001. SAL, saline; CL, *Curcuma longa*; DZP, diazepam; PTZ, pentylenetetrazol; HPC, hippocampus. Scale bar = 200 μm. CA1 and CA3: scale bar = 50 μm.

The quantification of the Nissl-stained neuron-like cells in the CA1 and CA3 regions of the hippocampus (Figure 6A) indicated that the animals pretreated with saline that received PTZ on the fifth day suffered a significant reduction in the number of neuron-like cells in the CA1 region [*F*_(7, 64)_ = 226.9; *p* < 0.0001; SAL + (SAL and CL and DZP and CL + DZP) vs. SAL + PTZ: *p* < 0.0001; Figure 6B]. Although pretreatment with both *C. longa* (SAL + PTZ vs. CL + PTZ: *p* < 0.0001) and DZP (SAL + PTZ vs. CL + PTZ: *p* < 0.0001) resulted in the significant preservation of these cells, and *C. longa* performed significantly better than DZP (*p* < 0.0001), the combination of these two substances (CL/DZP) was the best pretreatment for the preservation of the neuron-like cells (SAL + PTZ vs. CL/DZP + PTZ: *p* < 0.0001; CL/DZP + PTZ vs. CL + PTZ: *p* < 0.0001; CL/DZP + PTZ vs. CL + PTZ: *p* < 0.0001). This indicates a protective effect on the cells in the CA1 region of the hippocampus 7 days after the seizures.

In the case of the CA3 region of the hippocampus (Figure 6C), a significant change was observed only in the saline group that received PTZ on the fifth day [*F*_(7, 64)_ = 62.69; *p* < 0.0001], which indicates that a lack of adequate preventive treatment for seizures may result in the damage of this layer. All the pretreatments tested in the present study provided significant prevention of the loss of the neuron-like cells (*p* < 0.001; for all comparisons).

## Discussion

The results of the present study demonstrate that *C. longa* has anticonvulsant properties that are effective for the attenuation of PTZ-induced seizures. The data also showed that the combined application of *C. longa* with DZP decreased the seizure threshold and prevented the behavioral progression of the seizure, while also reducing the neuronal damage it causes.

Epilepsy is one of the most common disorders of the central nervous system that, when treated incorrectly or when the patient is resistant to the available medication, can impact the quality of life significantly (Sultana et al., 2021). Previous studies have shown that the recurrence of epileptic events may eventually have major degenerative effects that are also associated with a cognitive and behavioral decline. Some antiepileptic drugs may also cause harmful changes in the brain, which reinforces the need for the development of new treatments that can reduce the brain damage and minimize the side effects.

*C. longa* is widely cultivated in Asia, where it is a part of the traditional approach for the treatment of a variety of health problems, including gastrointestinal disorders, pain, and even epilepsy (Touhidi et al., 2018). While its mechanism of action is still unclear, some studies have found evidence of the modulation of the GABA receptors, which increases the synthesis of this neurotransmitter, reduces the activity of acetylcholinesterase, and inhibits the catecholaminergic and oxidative stress mechanisms (Aboul Ezz et al., 2011; Reeta et al., 2011; Vijayakumar et al., 2018). Other studies have also shown that *C. longa* reduces the activity of the glutamate receptors and contributes to the intracellular homeostasis of calcium (Noor et al., 2012).

Despite the evidence of its protective effects, the therapeutic potential of curcumin is limited by its poor bioavailability, given its reduced absorption and limited passage through the blood–brain barrier (BBB) (Tsai et al., 2011). These authors demonstrated that purified curcumin crosses the BBB at lower concentrations than when transported by nanoparticles. Technologies that facilitate the transport of curcumin to the brain, including nanocarriers and polymeric nanoparticles, are currently under investigation (Tsai et al., 2011; Askarizadeh et al., 2020). Given this, one of the limitations of the present study is the lack of the definition of the amount of curcumin that crossed the BBB.

The present study showed that pretreatment for 4 days with *C. longa* alone or in combination with DZP was able to reduce the duration of seizures. Saha et al. (2016) and Haghighizad et al. (2017) obtained similar results showing that treatment for at least 2 weeks with a minimum dose of 100 mg/kg of *C. longa* delayed the onset time and duration of tonic-clonic PTZ-induced seizures. Other studies have also corroborated these findings. Mehla et al. (2010) showed that curcumin caused a significant increase in the latency to the onset of seizures and reduced the mortality caused by the seizures induced by the repeated administration of a subconvulsant dose of PTZ. The present study obtained similar results through pretreatment with curcumin, even after the administration of only one dose of PTZ. This indicates that pretreatment or continuous treatment with curcumin may help shorten the duration of seizures, and it may be represented as a potential option for the treatment of epilepsy.

Some previous studies have also shown that the combination of *C. longa* with other antiepileptic drugs, such as sodium valproate, at a lower dose, may have a similar effect to the drug when administered alone (Aboul Ezz et al., 2011; Reeta et al., 2011; Noor et al., 2012). The present study showed that *C. longa* associated with DZP elicited a better response than either drug administered alone. These findings are extremely important, because the combination of *C. longa* with an AED may permit the reduction of the dose, which may, in turn, reduce its side effects.

The EEG trace of the seizures induced by PTZ had an amplitude of 0.3 mV, with high amplitude spike-waves, which were attenuated by the administration of *C. longa*. Orellana-Paucar et al. (2012) and Jiang et al. (2015) obtained similar results in which the use of curcumin, the principal biologically active component of *C. longa*, reduced the abnormal brain activity induced by the seizure. It is important to note that PTZ-induced seizures that can be extremely harmful and can cause hippocampal damage, especially in the CA1 and CA3 regions, may result in short- or long-term cognitive deficits (Kaur et al., 2014; Hashemian et al., 2017). The present study showed that the pretreatment, either with *C. longa* alone or in combination with DZP, reduces the damage in the hippocampus of rats, which is consistent with the previous studies that have demonstrated the potential protective properties of this substance.

It is interesting to note that epileptiform activity can be observed in almost 100% of surface EEGs, which can thus be used to predict possible brain injuries (Janszky et al., 2005; Singla et al., 2020). Although only a single pair of electrodes was used in the present study, which may be vulnerable to the influence of early motor signals, as well as the scalp and cerebrospinal fluid (Beleza and Pinho, 2011), which may limit spatial accuracy in comparison with multichannel systems, Johnstone et al. (2012) and Hemington and Reynolds (2014) validated this approach for EEG recording and diagnosis.

An increase in the delta and beta bandpower may reflect electrical alterations in the temporal and extratemporal lobes (Rosenow et al., 2015), and may also be present in other vascular diseases of the central nervous system (Ferreira et al., 2021). The present study showed that all three pretreatments (*C. longa*, DZP, or *C. longa*/DZP) reduced the bandpower of the delta and beta waves, which indicates that the seizure was controlled and brain damage was reduced. This indicates that *C. longa* may play a protective role, in particular, in the cells of the hippocampus that are highly sensitive to electrical and inflammatory disorders, and may become atrophied moderately or severely if left untreated.

Even so, the exact mechanisms through which the anti-inflammatory properties of *C. longa* are implemented are still unknown, although some authors have reported that it upregulates genes related to the anti-inflammatory cytokines and reduces the expression of pro-inflammatory cytokines, such as IL-1β and TNF-α (Hashemian et al., 2017; Yin et al., 2018). One other potential mechanism, described by Peng et al. (2021), is the inhibition of the expression of the iNOS gene by *C. longa* that interferes with the nitric oxide synthase pathway. Other studies have demonstrated the potential of *C. longa* for the protection of the hippocampal cells against electrical disturbances (Kaur et al., 2015; Hashemian et al., 2017), which is consistent with the findings of the present study, given the observed attenuation of the damage caused by PTZ in the CA1 and CA3 regions. These authors have also reported that *C. longa* inhibits the activation of astrocytes and microglia during electrical disturbances (Kaur et al., 2015; Hashemian et al., 2017).

Overall, the results of the present study indicate that *C. longa* has considerable potential for the control of the seizures and cell damage induced by PTZ, and that the association of this substance with DZP may represent a valuable approach for the treatment of epilepsy, thereby increasing the therapeutic options available to the patients. However, further research will be needed to better define the signaling pathways that determine the protective properties of *C. longa*.

## Data Availability Statement

The raw data supporting the conclusions of this article will be made available by the authors, without undue reservation.

## Ethics Statement

The animal study was reviewed and approved by the Ethics Committee on Use of Animals.

## Author Contributions

CN and LF performed the experiment and drafted the manuscript. ALMS, ABNS, JR, and LT conducted the bioinformatic analysis and interpreted the results. JA, DA, AH, BG, and BC performed the histological analyses. MH and DL reviewed and edited the manuscript. All authors contributed to the manuscript revision, and read and approved the submitted version.

## Conflict of Interest

The authors declare that the research was conducted in the absence of any commercial or financial relationships that could be construed as a potential conflict of interest.

## Publisher’s Note

All claims expressed in this article are solely those of the authors and do not necessarily represent those of their affiliated organizations, or those of the publisher, the editors and the reviewers. Any product that may be evaluated in this article, or claim that may be made by its manufacturer, is not guaranteed or endorsed by the publisher.
